# Demographic and Geospatial Analysis of Buprenorphine and Methadone Prescription Rates

**DOI:** 10.7759/cureus.25477

**Published:** 2022-05-30

**Authors:** Nicholas J Peterman, Peggy Palsgaard, Aksal Vashi, Tejal Vashi, Bradley D Kaptur, Eunhae Yeo, Warren Mccauley

**Affiliations:** 1 Medicine, Carle Foundation Hospital, Urbana, USA; 2 Medicine, Carle Illinois College of Medicine, Champaign, USA; 3 Statistics, Social & Scientific Systems, Atlanta, USA; 4 Anesthesiology, Carle Foundation Hospital, Urbana, USA

**Keywords:** rural health, addiction, methadone, buprenorphine, opioid use disorder

## Abstract

Background

The medical community continues to seek to understand both the causes and consequences of opioid use disorder (OUD). The recent 2019 public release of the Automation of Reports and Consolidated Orders System (ARCOS) database from the years 2006 to 2012 provides a unique opportunity to analyze a critical period of the opioid epidemic with unprecedented data granularity.

Objectives

This study aims to use the ARCOS dataset to (1) determine significant contributory variables to opioid overdose death rates, (2) determine significant contributory variables to the relative prescription of buprenorphine and methadone, and (3) evaluate the existence of statistically significant geospatial clusters in buprenorphine and methadone prescription rates.

Methods

This study utilizes multiple databases, including the Centers for Disease Control and Prevention (CDC) Wide-ranging Online Data for Epidemiologic Research (WONDER), the Drug Enforcement Administration (DEA) prescription drug data, and the United States (US) Census demographics, to examine the relationship between the different treatments of OUD. Linear regressions are used to determine significant contributory factors in overdose rate and the buprenorphine-to-methadone ratio. Geospatial analysis is used to identify geographic clusters in opioid overdoses and treatment patterns.

Results

Methadone prescriptions, racial demographics, and poverty were found to significantly correspond to opioid overdose death rates (p < 0.05). Buprenorphine prescriptions were not found to be significant (p = 0.20). Opioid overdoses, metro character, racial categorization, and education were found to significantly correspond to the ratio of buprenorphine to methadone prescribed (p < 0.05). Cluster analysis demonstrated different geospatial distributions in the prescriptions of buprenorphine and methadone (p < 0.05).

Conclusion

Historically, methadone prescriptions have been higher in areas with high overdose rates. Buprenorphine and methadone prescribing patterns have historically demonstrated different geographic trends.

## Introduction

The opioid epidemic and the increased prevalence of opioid use disorder (OUD) have had a widespread impact on families and communities across the United States (US) since the late 1990s [1]. In 2019 alone, approximately 10.1 million people were estimated to have misused or abused opioids across the country [2]. In response to the opioid crisis, the Food and Drug Administration (FDA) has approved three drugs for the treatment of OUD (methadone, buprenorphine, and naltrexone), and the 2020 American Society of Addiction Medicine (ASAM) guidelines state that all patients should have access to all FDA-approved medications [3,4].

The ease of prescribing these medications varies. The Drug Addiction Treatment Act of 2000 allowed physicians to obtain waivers for prescribing buprenorphine to patients suffering from OUD in any clinical setting [5]. Unfortunately, while buprenorphine access has steadily increased, it has not done so rapidly enough to meet demand. As of 2019, rural counties in the US are still disproportionately lacking access to buprenorphine prescribers [5]. While the removal of some restrictions on the buprenorphine X-waiver was revisited in early 2021, there remain moderate institutional barriers to its widespread use. Specifically, the easement of certification restrictions is only beneficial for providers intending to use buprenorphine in the care of fewer than 30 patients [6,7]. Access to methadone as a treatment for OUD has historically not been covered by Medicare and has been more difficult to obtain; it can only be administered in opioid treatment programs (OTPs) and acute care settings in limited situations [3]. The reasoning behind these restrictions is multifaceted; however, methadone’s history of abuse is a noteworthy factor. Previous works have arrived at conflicting conclusions with respect to methadone’s benefits compared to buprenorphine, naltrexone, and abstinence [8-11].

In 2018, the US federal government passed the Substance Use-Disorder Prevention That Promotes Opioid Recovery and Treatment for Patients and Communities Act (SUPPORT); this law cleared the way for methadone to be used for the treatment of OUD under Medicare for the first time [12]. As a result, it can be expected that communities across the US are experiencing a wave of increased access to methadone. It is valuable to identify potential risks associated with increased methadone access and predict the impacts of this adjustment. While there are no data available since the passing of the SUPPORT Act, this study will conduct a retrospective analysis of methadone prescription rates, buprenorphine prescription rates, demographic trends, geospatial trends, and overdose deaths using publicly available datasets from the Drug Enforcement Administration (DEA), the Centers for Disease Control and Prevention (CDC), and the US Census Bureau to investigate how treatments for OUD vary across the country and correlate with county-level overdose deaths. The null hypothesis of this study is threefold: (1) the demographic variables of interest do not have a statistically significant relationship with the opioid overdose rate; (2) the demographic variables of interest do not have a statistically significant relationship with the ratio of buprenorphine-to-methadone prescriptions; and (3) the prescriptions of buprenorphine and methadone are geographically random without statistically significant clustering present.

## Materials and methods

Database sources

In an attempt to provide a more robust analysis of the nationwide trends that exist within the opioid crisis, this study utilizes multiple databases including the DEA prescription drug data, the US Census demographics, and the CDC Wide-ranging Online Data for Epidemiologic Research (WONDER) data. The Automation of Reports and Consolidated Orders System (ARCOS) is a DEA monitoring system that overlooks the flow of controlled substances and accumulates these transactions into reports [13,14]. In 2019, the Washington Post released a dataset that included the opioid subset of the DEA ARCOS database from 2006 to 2012 [15]. This DEA ARCOS database was used to extract all instances of buprenorphine and methadone being prescribed between 2006 and 2012 in the contiguous United States. These data are not limited to OTPs, but instead reflect every legitimate opioid prescription made in the US. The CDC WONDER database provides a large quantity of epidemiologic data, from which we obtained the age-adjusted overdose death rate per county as the closest proxy for the opioid death rate. This substitution was made with the knowledge that opioids account for approximately 66% of all overdose deaths [16-18]. Finally, both of these databases were related via US Census data from 2006 to 2012.

This study utilizes a Python-based script for database building and cleaning, as well as GeoDa, which is a statistical map-based graphing software. GeoDa was used to chart the demographic, geographic, and socioeconomic trends and their relationship to overdose deaths across 2917 US counties that had methadone and buprenorphine prescribed at least once in 2006-2012. Of the total counties in the contiguous United States, 92.8% were included in the analysis. A similar technique of utilizing the DEA ARCOS database and the CDC WONDER database alongside additional demographic data has been utilized in a previous study providing descriptive statistics on opioid use distributions and healthcare access in the US [19]. Of the three pharmacological treatments approved for OUD, this paper will focus on methadone and buprenorphine, as they are both opioid agonists with abuse potential. Naltrexone will not be included as it is an opioid antagonist, and thus is not a candidate for abuse. Naltrexone prescription data were also of limited availability in our dataset.

To examine the relationship between urban and rural areas, the United States Department of Agriculture (USDA) Economic Research Service’s (ERS) Rural-Urban Continuum Codes from 2013 were used [20]. The Rural-Urban Continuum Codes provide a range of ratings that provide a more specific description of a community versus a binary of “metro” or “non-metro.” The descriptive values are on a sliding scale of 1 to 9, with 1 being assigned to counties in a metro area with a population of one million or more, and 9 being assigned to counties with a population of less than 2,500 not adjacent to a metro area. The percentage that completed certain educational milestones (General Educational Development (GED), college degree, etc.) was also available with this dataset and was included as a demographic variable. The rural-urban continuum was included, as multiple studies have found relationships between high overdose rates and metropolitan rates [21,22].

CDC WONDER data for county-level opioid deaths from 2006 to 2012 were collected in the form of age-adjusted opioid death rate per 10,000 population. County-level areas with less than 11 but not zero deaths occurring over the requested time period or with an age-adjusted rate less than 0.02 were suppressed per CDC public use protocol [23]. To fill in any county-level data gaps the suppressed values created, the average state-level value was used to replace suppressed values in their respective counties [24].

Standardizing OUD treatments

To look at data for maintenance treatment of OUD, the drug data were filtered by listed mode of delivery (i.e. tablet, syringe, or sublingual) to only those types that were approved for maintenance treatment. First, buprenorphine is FDA approved for the treatment of OUD in the delivery methods of film (buccal and sublingual), injection, and tablet. However, injections are only used for initiation, rather than maintenance, and were thus ignored. Only film (buccal and sublingual) and tablet delivery methods were included in this dataset. Methadone, however, is FDA approved for the treatment of OUD in tablet and oral concentrate. No other delivery methods were found in the database, so no methadone samples were removed based on this criterion. There were 458 different prescription names due to variations in naming conventions by pharmacies. All prescription names were examined manually and checked to ensure that they were either methadone or buprenorphine. Current databases were checked for names [25].

Buprenorphine and methadone cannot be directly compared to each other as their relative potencies vary. For example, using the Centers for Medicare and Medicaid Services conversion factor, buprenorphine film has a morphine equivalent of 30, while methadone (>60 mg) has a morphine equivalent of 12 [26]. To compare, it was necessary to convert their dosage provided to a “days equivalent.” The average maintenance dose of the respective drugs was used to create a buprenorphine days equivalent (BDE) and a methadone days equivalent (MDE). The calculation was as follows:

1. $BDE= \frac{ dosage\, unit \times unit\, strength}{16\, mg\, per\, day}$.

2. $MDE= \frac{ dosage\, unit \times unit\, strength}{100\, mg\, per\, day}$.

For example, a prescription of 500 mg of methadone would be approximated to a five-day dosage as follows:

3. $MDE= \frac{ (500\, mg)\times(1\: mg)}{100\, mg\, per\, day}= 5\, days$.

A number of methadone prescriptions were missing dosage because they were given in a liquid form and the “dosage unit” that was utilized above is often not filled out in this case. However, the unit “CALC_BASE_WT_IN_GM” is mandated to be reported for each prescription. The methadone anhydrous conversion factor reported in the ARCOS Registrant Handbook was used (methadone = 0.8946; buprenorphine = 0.9275) [13]. Utilizing an anhydrous conversion factor, this can be converted to a “dosage unit” as such:

4. $Dosage\, unit\times unit\, strength= \frac{calculated\, base\, weight\, in\, grams\, (1000\, mg/g)}{anhydrous\, conversion\, factor}$.

BDE and MDE represent the days provided by the prescription listed in the data and are comparable to each other as an approximate amount of buprenorphine and methadone given for the treatment of OUD. Next, these values were scaled to the number of days per 10,000 people using the population of each county.

Finally, each calculation was logarithmically transformed to arrive at a standard form that would be resistant to skew before multivariate analysis. Scale-location plots of each regression were created to confirm homoscedasticity and affirm the validity of the log transform.

Analysis tools

The dataset was initially analyzed in Python before being exported to GeoDa, a spatial analysis program, for further categorization of potential geospatial clustering and for improving the multivariate regression by utilizing spatial weighting [27]. Although multivariate regressions are critical for controlling for confounding variables, cluster analysis can identify and prove the statistical significance of complex spatial patterns in the variables that cannot be condensed linearly or logarithmically.

Moran’s I is a statistical measure of how similar an object’s attributes are to its neighbors [28]. It is calculated at a global level for a dataset utilizing each object in the respective dataset. In our study, the object is a Federal Information Processing Standards (FIPS) area code corresponding to a US county and the attributes are the descriptive statistics of that area code, such as race, death rate, and income. The global Moran’s I will give the equivalent of a p-value for each attribute in the data frame to reject the null hypothesis that the attribute is spatially random, and instead choose the alternative hypothesis that clustering is present. While this is an excellent statistic by itself, it does not indicate where these clusters are but simply indicates that they exist. Thus, a local indicator of spatial association (LISA) statistic is used by calculating local Moran’s I for each object, with their average being equivalent to the global Moran’s I for the entire dataset [29]. This local Moran’s I can then be mapped using the corresponding FIPS area code. From there, the spatial clusters can be further broken down into four groups of spatial outliers: High-High, High-Low, Low-High, and Low-Low. These conventional naming groups reference the object’s relationship to its neighbors relative to the mean of the overall attributes of interest. Local Moran’s I was conducted on the buprenorphine-to-methadone ratio and opioid death rate individually. Map-based visualizations of both the clustering and variable percentiles were created for visualization of the aforementioned variables and analysis.

## Results

**Figure 1:**
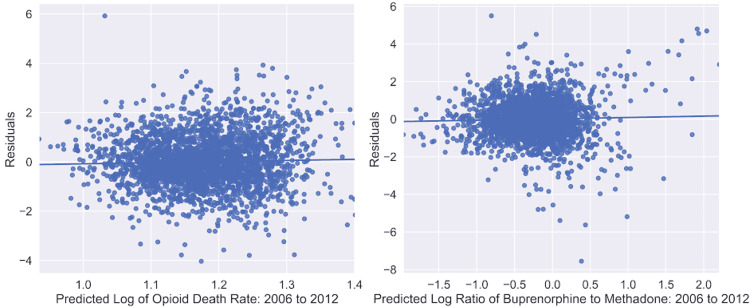
Scale-location plots

Separate geospatially weighted, linear regressions were conducted for the dependent variables of overdose deaths and buprenorphine-to-methadone ratio using the data from 2006 to 2012. Given that the initial data had to be logarithmically transformed, there are concerns about homoscedasticity and linearity. Scale-location plots of each regression (Figure 1) confirm homoscedasticity and linearity, affirming the validity of the log transform. Nineteen variables, which spanned demographic parameters as well as prescription rates, were initially utilized as independent variables (Table 1). Independent variables with p-values ≥ 0.05 were removed using a manual implementation of stepwise optimization. The results of the regressions are presented in Tables 2, 3.

**Table 1:** Variables initially used in geospatial linear regressions GED, General Educational Development.

White (%)
Black (%)
Asian (%)
Hispanic (%)
Population (2006-2012)
Population density
Metro (binary value 0, 1)
Urban (binary value 0, 1)
Poverty (%)
Median household income
Rural-Urban Continuum Code (integer 1-9, 1 = most urban, metro <= 3)
Without GED (%)
With only GED (%)
Some college (%)
College degree (%)
Overdose death rate (2006-2012)
Buprenorphine/methadone ratio (2006-2012)
Buprenorphine days (2006-2012)
Methadone days (2006-2012)

**Table 2:** Geospatially weighted linear regression results for overdose deaths from 2006 to 2012 r^2^ = 0.640; df = 2907.

Variable	Coefficient	Std. error	z-value	Probability
Constant	0.832	0.048	17.417	<0.001
Log population density	0.057	0.005	10.538	<0.001
Log methadone days	0.017	0.005	3.583	<0.001
% White	0.002	<0.001	4.680	<0.001
% Black	-0.001	<0.001	-3.124	0.002
% Asian	-0.006	0.002	-4.004	<0.001
% Hispanic	-0.003	<0.001	-9.744	<0.001
% College degree	-0.004	<0.001	-11.008	<0.001
% Poverty	0.007	0.001	11.341	<0.001
Log buprenorphine days	0.005	0.004	1.282	0.200

**Table 3:** Geospatially weighted linear regression results for buprenorphine-to-methadone ratio from 2006 to 2012 r^2^ = 0.632; df = 2911.

Variable	Coefficient	Std. error	z-value	Probability
Constant	-2.236	0.084	-26.695	<0.001
Log buprenorphine days	0.531	0.011	46.431	<0.001
% without a high school diploma	0.005	0.002	3.201	0.001
% Black	-0.003	0.001	-3.712	<0.001
Log opioid overdoses	-0.233	0.061	-3.821	<0.001
Metro	-0.064	0.018	-3.653	<0.001

The r^2^ value of the overdose regression was high (r^2^ = 0.640), indicating a high proportion of the variance in overdose death outcomes was able to be explained by the independent variables. The r^2^ value of the buprenorphine-to-methadone ratio regression was similarly strong (r^2^ = 0.632), indicating that the local ratio of buprenorphine prescription rates to methadone prescription rates is able to be strongly modeled by the variables of interest.

For the overdose deaths regression, the highly significant variables included racial categorizations, the population density, the percentage in poverty, and the percentage with a college degree. Of note, the number of methadone prescriptions was positively significantly correlated with overdose deaths (p < 0.001). Buprenorphine prescriptions were positively correlated but did not have statistical significance (p = 0.20). Of the racial categorizations, an increased white percentage of a given county was associated with an increase in opioid deaths (p < 0.001). Increased Black (p = 0.002), Asian (p < 0.001), and Hispanic (p < 0.001) representation was associated with a decrease in opioid deaths. As the percentage with a college degree increased, the opioid deaths were predicted to decrease (p < 0.001) in a given area (Table 1).

For the buprenorphine-to-methadone ratio regression, statistically significant variables in predicting the ratio included the percentage without a high school diploma, the percentage of the population that was Black, the number of opioid overdoses, and the metro character of the county. The percentage without a high school diploma had a positive coefficient in the model (p = 0.001), suggesting in those areas, more buprenorphine than methadone was prescribed (relative to the national average). The percentage that was Black, the opioid overdoses, and metro character had negative coefficients (p < 0.001), suggesting that increases in those variables corresponded to areas where more methadone than buprenorphine was prescribed (relative to the national average) (Table 2).

**Figure 2:**
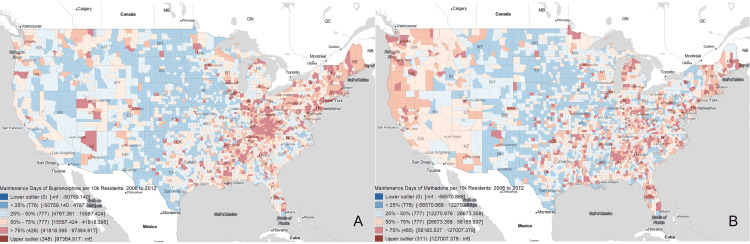
Prescription distribution of opioid use disorder pharmacotherapy (A) Equivalent buprenorphine maintenance days prescribed per 10,000 people from 2006 to 2012 in the United States. (B) Equivalent methadone maintenance days prescribed per 10,000 people from 2006 to 2012 in the United States.

Figure 2 provides nationwide buprenorphine and methadone prescription distribution visualizations for each of the 2917 FIPS area codes. These box maps show a sliding color scale of equivalent maintenance days prescribed per 10,000 people for buprenorphine and methadone independently. The highest prescription rates of buprenorphine follow a trend very similar to that of opioid deaths, concentrated heavily in Mid-Atlantic Appalachian states with other upper outliers spread across the Eastern US and a few in the Western US. Methadone prescription rates follow a less defined pattern, with upper outliers spread across the Eastern US and a generalized higher level of utilization across the Western and Southwestern US.

**Figure 3:**
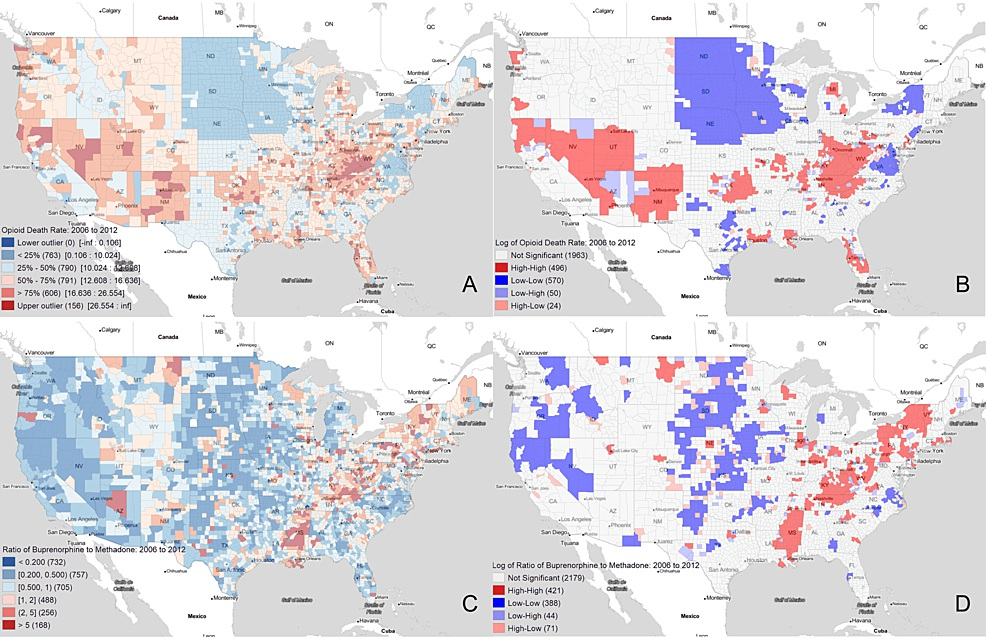
Cluster analysis of opioid use disorder pharmacotherapy (A) Box map visualization of age-adjusted overdose rate per 10,000 people from 2006 to 2012. (B) Local Moran's I plot demonstrating clusters of age-adjusted overdose rates. (C) Box map visualization of buprenorphine-to-methadone ratio from 2006 to 2012. (D) Local Moran's I plot demonstrating clusters of buprenorphine-to-methadone ratio.

Figure 3 provides local Moran’s I cluster analysis in opioid and drug prescription ratio data. The geospatial maps of age-adjusted overdose death rate per 10,000 people and Moran’s I cluster analysis show large areas of upper outliers in the Mid-Atlantic Appalachian and the Southwestern US with areas of very low overdose deaths rates in the northeast and northern Midwest. As for the analysis of the buprenorphine-to-methadone prescription rate ratio, the box map visualizes areas of upper outliers across Appalachia and a smaller number of intermittent communities across the Western US. The local Moran’s I map shows clustering of higher rates of buprenorphine-to-methadone prescription rate ratios surrounded by areas of lower buprenorphine-to-methadone prescription rate ratios that do not align with areas of highest population density; instead, they follow the same trends described in the previous figure.

## Discussion

The opioid epidemic is a complex public health crisis that many previous researchers have dedicated substantial effort toward understanding. The 2019 public release of the ARCOS dataset from the years 2006 to 2012 [13,14] represents a new opportunity to draw insightful conclusions from a critical period of the opioid epidemic. In contrast to other national databases, including Medicare datasets that have been popular sources for previous studies, the ARCOS database represents the most complete public release of opioid usage data thus far. Using this and other datasets, this study categorizes overdose death rates in relation to the treatments used for OUD in an effort to elucidate trends in their demographic and geospatial relationships.

Of the 2917 FIPS codes analyzed, 70% prescribed more methadone than buprenorphine from 2006 to 2012 and 49% prescribed more methadone than buprenorphine in 2012 alone (Figure 2). Both methadone and buprenorphine prescription rates increased between 2006 and 2012 but buprenorphine prescription rates increased more substantially. Overall, between 2006 and 2012, buprenorphine was prescribed 2.2 times more often than methadone. More counties prescribed methadone more often, but in the areas that prescribed buprenorphine, buprenorphine was prescribed at such a high rate that the nationwide prescribing trend was reversed. These findings together suggest that buprenorphine prescription rates seem to have a more distinct geographical prescription pattern. In contrast, methadone prescription rates appear to follow a diffuse geographic pattern of prescription.

Geospatial analysis mapping of CDC overdose data and prescription data to specific FIPS area codes allows for additional insight. Figure 2 provides box map visualizations of the total buprenorphine and methadone prescription rates per 10,000 people for US counties between 2006 and 2012. Buprenorphine prescriptions follow a trend of higher concentrations in the Mid-Atlantic Appalachian region, the New England region, and a few areas in the Western and Southwestern US. On the other hand, methadone seems to be prescribed at higher rates across the entire eastern half of the country. Methadone prescriptions also appear more prominent on the West Coast.

Figure 3 provides box maps and Moran’s I cluster analyses both for overdose deaths and the buprenorphine-to-methadone prescription ratio for US counties between 2006 and 2012. Using the box map and Moran’s I cluster analysis for overdose deaths (Figures 3A, 3B), two main clusters of overdose deaths exist in the US: one in the Southwest, and one in the Mid-Atlantic Appalachian region (as well as a third slightly less definitive cluster in Oklahoma and the Four State Area). The box map and Moran’s I cluster analysis for the buprenorphine-to-methadone ratio (Figures 3C, 3D) demonstrate High-High clusters (meaning buprenorphine is relatively favored) throughout the Mid-Atlantic Appalachian region. Similarly, they demonstrate Low-Low clusters (meaning methadone is relatively favored) throughout the Great Plains and West Coast. This provides statistical support to the visually observed trends mentioned earlier.

Geospatially weighted, linear regression served as the first step in identifying significant predictors of any given FIPS area code’s overdose rate and buprenorphine-to-methadone prescription ratio. First, regressions conducted on data from 2006 to 2012 demonstrated that overdoses were predicted strongly by the racial makeup of a county (Table 2). These findings are consistent with others that have found the race to be a differentiating factor in overdose rate [30]. Previous spatiotemporal analysis (limited to the state of Ohio) also found the race to be an important factor in overdose deaths across counties [31]. The race has many distinct ties to social determinants of health and likely encompasses a variety of factors that may not have been directly accounted for in the geospatial regression [32-34].

Additional geospatial regressions were conducted with the buprenorphine-to-methadone ratio as the dependent variable. This analysis showed that the number of opioid overdoses, the metropolitan character of a county, and the percentage of Black persons in a county were all negatively correlated with this ratio (Table 3). The negative correlation in the number of opioid overdoses with the buprenorphine-to-methadone ratio provides additional support for the notion that methadone prescriptions are higher in areas with higher overdose deaths. The significance of the percentage of Black persons in this ratio suggests that there may be racial disparities involved in the differential accessibility of these medications.

Given the data obtained from multivariate demographic analysis and geospatial analysis, our hypotheses can be revisited. Our regressions demonstrate that both the number of overdose deaths and the buprenorphine-to-methadone ratio can be strongly modeled by specific demographic factors (Tables 2, 3). Additionally, our geospatial analysis using the Moran’s I coefficient demonstrates statistically significant clustering in specific regions of the US (Figure 3). Thus, a key takeaway is that the OUD crisis is causing disproportionate suffering and loss of life in specific geographic regions of the US. Our geospatially weighted analysis was subsequently justified in that these geographic trends would have been missed in a purely national-level analysis.

Limitations

This study was not without weaknesses. Our data were based on overdose deaths per county, rather than opioid deaths per county. Unfortunately, the CDC WONDER database only provides data on overall overdose rates as opposed to opioid overdose rates. We decided to still implement these data given that opioids account for over 66% of overdose deaths [16-18]. We present these results under the assumption that the rate of opioid overdose deaths as a percentage of total overdose deaths would be approximately equivalent across the country. Additionally, the CDC suppresses death counts in area codes with less than 11 deaths. In areas where reported deaths were less than 11 (but nonzero), the statewide death rates were substituted. This may have led to some geographic artifacts in the form of hotspots in the geospatial cluster analysis in areas that were relatively spared from the opioid epidemic. However, given that the scope of this paper is analyzing regional geographic trends across the country, the existence of local hotspots on the level of individual counties would not be expected to affect our overall conclusions.

## Conclusions

The importance of this work is to further understand and characterize the opioid epidemic and how those suffering from OUD are receiving treatment across the country. Methadone prescriptions have historically significantly correlated with higher overdose rates; though, the same cannot be said for buprenorphine based on the results of this study. Buprenorphine and methadone have demonstrated somewhat different prescription trends, with methadone demonstrating a more diffuse spread. As the legislative landscape of treatment of OUD shifts in the upcoming years, it will be important to understand these historical trends for policymaking guidance.
